# A perspective on 16S rRNA operational taxonomic unit clustering using sequence similarity

**DOI:** 10.1038/npjbiofilms.2016.4

**Published:** 2016-04-20

**Authors:** Nam-Phuong Nguyen, Tandy Warnow, Mihai Pop, Bryan White

**Affiliations:** 1 Carl R. Woese Institute for Genomic Biology, University of Illinois at Urbana-Champaign, Champaign, IL, USA; 2 Department of Bioengineering, University of Illinois at Urbana-Champaign, Champaign, IL, USA; 3 Department of Computer Science, University of Illinois at Urbana-Champaign, Champaign, IL, USA; 4 Center for Bioinformatics and Computational Biology, University of Maryland, College Park, College Park, MD, USA

## Abstract

The standard pipeline for 16S amplicon analysis starts by clustering sequences within a percent sequence similarity threshold (typically 97%) into ‘Operational Taxonomic Units’ (OTUs). From each OTU, a single sequence is selected as a representative. This representative sequence is annotated, and that annotation is applied to all remaining sequences within that OTU. This perspective paper will discuss the known shortcomings of this standard approach using results obtained from the Human Microbiome Project. In particular, we will show that the traditional approach of using pairwise sequence alignments to compute sequence similarity can result in poorly clustered OTUs. As OTUs are typically annotated based upon a single representative sequence, poorly clustered OTUs can have significant impact on downstream analyses. These results suggest that we need to move beyond simple clustering techniques for 16S analysis.

## Introduction

As the 16S rRNA gene is universally present across bacteria, is highly conserved, and can be easily amplified using universal primers, environmental microbial analyses are often performed using 16S rRNA amplicon sequencing. Although the 16S rRNA gene is highly conserved, there are nine hypervariable regions that can be used to distinguish between different organisms. The typical pipeline for 16S amplicon analyses starts with using primers designed to amplify the hypervariable regions of the 16S rRNA gene (typically the V1–V3 region or the V3–V5 region). Sequences are clustered into bins called ‘Operational Taxonomic Units’ (OTUs) based upon similarity. Typically, the similarity between a pair of sequences is computed as the percentage of sites that agree in a pairwise sequence alignment. A common similarity threshold used is 97%, which was derived from an empirical study that showed most strains had 97% 16S rRNA sequence similarity.^1^ From the OTU cluster, a single sequence is selected as a representative sequence. The representative sequence is annotated using a 16S classification method,^2,3^ and all sequences within the OTU inherit that same annotation. Several pipelines have been developed to perform the entire 16S analysis from end to end, including QIIME^4^ and MOTHUR.^5^

One of the largest benefits of OTU clustering is computational. Typically, a 16S amplicon analysis can have millions of reads, however, this may result in only thousands of OTUs. Downstream analyses, such as multiple sequence alignment (MSA) or phylogeny estimation, become more tractable when working on the representative sequence set. Thus, clustering allows for rapid analysis of amplicon data sets.

However, there have been many criticisms with using percent sequence similarity to define OTUs.^6–8^ First, the percent sequence similarity can overestimate the evolutionary similarity between pairs of sequences. For example, sequence similarity computed from pairwise alignments underestimates the number of substitutions compared with similarity computed from MSAs.^9^ In addition, the percent similarity is a nonevolutionary-based distance metric; it fails to take into account that multiple substitutions can occur at the same site.^10^ These studies suggest that the best practice for computing similarity between sequences is to use evolutionarily corrected distances based upon a MSA; however, typical analyses use uncorrected distances based upon pairwise sequence alignments.

Second, the 97% 16S rRNA sequence similarity threshold used to delineate species is only a rough approximation. For example, two different species may have 99% similar 16S sequences (such as *Bacillus globisporus* and *B. psychrophilus*^11^) or the same strain may have multiple copies of the 16S rRNA gene that differ by 5% for some regions (such as *Escherichia coli K12*^12^). Even using the hypervariable regions can still lead to ambiguity; Huse *et al.* found that 18% of the V3 region mapped to two or more rRNA sequences.^13^

This perspective paper focuses on the problems of using sequence similarity for defining OTUs. We will discuss three different dissimilarity metrics for quantifying the evolutionary distance between pairs of sequences:

- Pairwise alignment sequence dissimilarity (PSD): For a pair of sequences, an optimal pairwise alignment is computed and the dissimilarity is defined as the percentage of sites that disagree in the pairwise alignment. This is the traditional approach for estimating the similarity or dissimilarity between sequences.
- MSA-based sequence dissimilarity (MSD): All sequences in the data set are aligned into a single MSA. The dissimilarity between a pair of sequences is defined as the percentage of non-gapped sites that disagree in the induced pairwise alignment.
- Phylogenetic branch length distance (BLD): A maximum likelihood (ML) tree is estimated on the MSA of the sequences. The dissimilarity between a pair of sequences is defined as the total branch length distance on the ML tree between a pair of sequences.

Both the PSD and MSD are types of p-distances, which are distances based upon the proportion of sites that disagree between a pair of sequences. The p-distance does not make any corrections for multiple substitutions at the same site, for varying rates of evolution among sites, or for substitution rate biases. Thus, the p-distances are most useful when the amount of evolution between sequences is low; otherwise it will underestimate the total amount of evolution between sequences. The BLD, on the other hand, is an evolutionary distance that estimates the expected number of substitutions per site, and thus, more accurately reflects the total number of evolutionary events between pairs of sequences. Thus, although the PSD and MSD are directly comparable (both are p-distances), the units for the BLD are fundamentally different. However, the BLD can be used as a metric to determine how phylogenetically diverse an OTU can be.

Although BLD distances might better estimate the true evolutionary distances between sequences, PSD and MSD are more commonly used metrics. As a case study, we use a subset of the QIIME-based analyses from the Human Microbiome Project^14^ (HMP), which computed OTUs using PSD based upon a 97% sequence similarity threshold (corresponds to maximum PSD of 0.03). We take the V1V3 and V3V5 OTUs that were generated from the HMP and compute the MSD and BLD between the representative sequence and all remaining sequences within the OTU (PSD was excluded as we were unable to extract the PSD from the USEARCH results).

By examining the MSD and BLD metrics, we can compare the compactness of an OTU that was originally computed using PSD. By definition, all sequences within an OTU should be at most 3% dissimilar from the representative sequence (PSD<0.03). We will show that the compactness of the OTUs varies greatly across the taxonomic tree, and both MSD and BLD often exceed the 0.03 threshold for an OTU.

## Results and discussion

**Figure 1:**
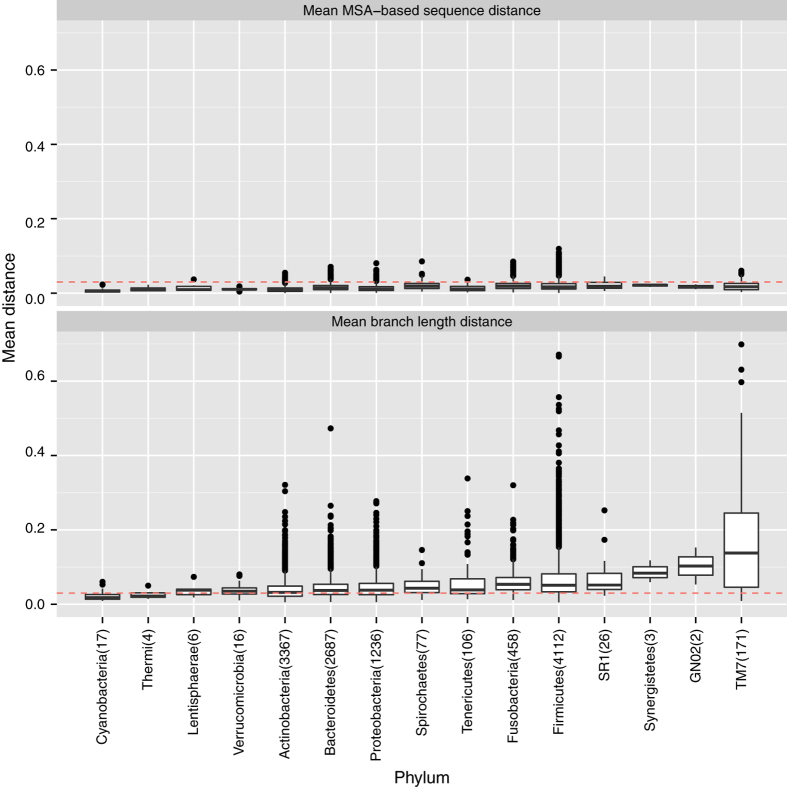
Box plots of the mean MSA and BLD across the different phyla for the V1V3 OTUs. We report the distribution of the mean MSA-based sequence dissimilarity and mean phylogenetic branch length distance between the representative sequence and all other sequences within an OTU for the V1V3 OTUs. We group the distributions according to the OTUs’ phylum-level annotations. The red line delineates a distance of 0.03. We report the total number of OTUs that belong to that phylum in parenthesis.

**Figure 2:**
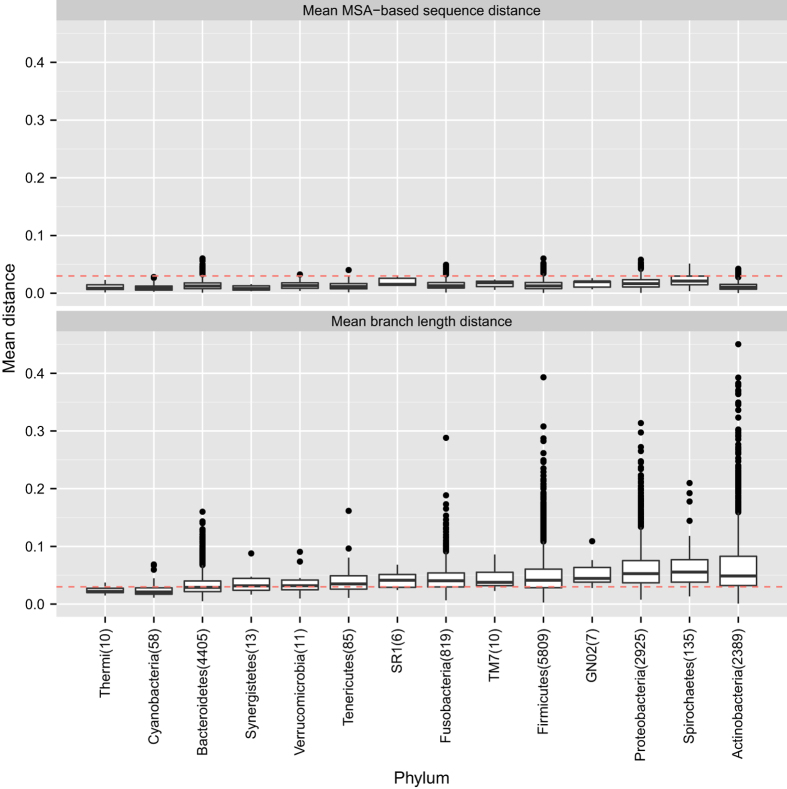
Box plots of the mean MSA and BLD across the different phyla for the V3V5 OTUs. We report the distribution of the mean MSA-based sequence dissimilarity and mean phylogenetic branch length distance between the representative sequence and all other sequences within an OTU for the V3V5 OTUs. We group the distributions according to the OTUs’ phylum-level annotations. The red line delineates a distance of 0.03. We report the total number of OTUs that belong to that phylum in parenthesis.

When we examined the OTUs that were generated using PSD, we found that many OTUs had a MSD or BLD that was >0.03 (see Figures 1 and 2). We found that 80.5% of the V3V5 OTUs had at least one sequence that was >3% dissimilar (MSD>0.03) from the representative sequence, and 2.7% of all sequences across all the V3V5 OTUs had a MSD >3% from the representative. In addition, 4.7% of the V3V5 OTUs were very diverse and had an average MSD>0.03.

Interestingly, the results for the V1V3 OTUs showed that the V1V3 OTUs were even more diverse compared with the V3V5 OTUs. With respect to the MSD, 84.4% of the V1V3 OTUs had at least one sequence that was >3% dissimilar from the representative, 6.4% of all the V1V3 sequences were >3% dissimilar from the representative, and 9.5% of all V1V3 OTUs had an average MSD >0.03.

When we classified every read within an OTU, we found that 12.9% of all the V3V5 OTUs and 19.8% of all the V1V3 OTUs contained sequences with annotations that differed from the representative sequence. For OTUs that had an average MSD of 0.03 or greater, that percentage rose to 13.5% for the V3V5 OTUs, and 26.2% for the V1V3 OTUs. Thus, OTUs that were more divergent were more likely to have multiple possible annotations, especially for the reads from the V1V3 regions.

The MSD still seemed to underestimate the total amount of evolution between sequences by an approximate factor of 3.4 for both the V1V3 and V3V5 reads: the average MSD and BLD for the V1V3 OTUs were 0.016 and 0.054 and the average MSD and BLD for the V3V5 OTUs were 0.014 and 0.049. We found that 78.9% of all V3V5 sequences and 81.2% of all V1V3 sequences had a BLD>0.03 from the representative. These observations suggest that BLD might be more sensitive in detecting evolutionarily divergence sequences.

Both the MSD and BLD varied greatly across the taxonomic tree (Figures 1 and 2). For example, both the V1V3 and V3V5 OTUs for the phylum Cyanobacteria were all very compact (mean MSD and BLD of 0.007 and 0.023 for V1V3 and mean MSD and BLD of 0.010 and 0.025 for the V3V5). The OTUs for the candidate phylum TM7, however, showed much greater variation and diversity in the V1V3 region: the mean MSD and BLD were 0.020 and 0.171 (more than three times the average BLD). Interestingly, although the V3V5 OTUs from the candidate phylum TM7 showed much lower diversity (the mean MSD and BLD were 0.016 and 0.046), the source of this discrepancy may be caused by sampling: only 5,776 V3V5 reads belonged to OTUs annotated as candidate phylum TM7 compared with a total 226,248 V1V3 reads.

We found that the MSD and BLD for individual reads were weakly correlated (Pearson’s correlation coefficients of 0.10 and 0.38 for the V3V5 and V1V3 regions). When we computed the correlation coefficients for the mean MSD and BLD for the OTUs, the Pearson’s correlation coefficients increased (0.41 and 0.63 for the V3V5 and V1V3 regions). We believe one of the reasons why these metrics are not more strongly correlated is because the MSD is a Hamming Distance (i.e., it counts raw differences), whereas the BLD is an evolutionary metric (i.e., it counts the expected number of substitutions per site using a phylogeny). The correlation between MSD and BLD might be more positively correlated if the MSD was corrected under a model of sequence evolution (i.e., such as a log-det distance model^15–17^ or a GTR distance model^18^).

For the remainder of this study, we present examples of different distributions for the MSD and BLD distances for selected V1V3 OTUs. We note that the observations about these selected V1V3 OTUs are not unique, and that OTUs with similar diversity can be found throughout both the V3V5 OTUs and the remaining V1V3 OTUs.

**Figure 3:**
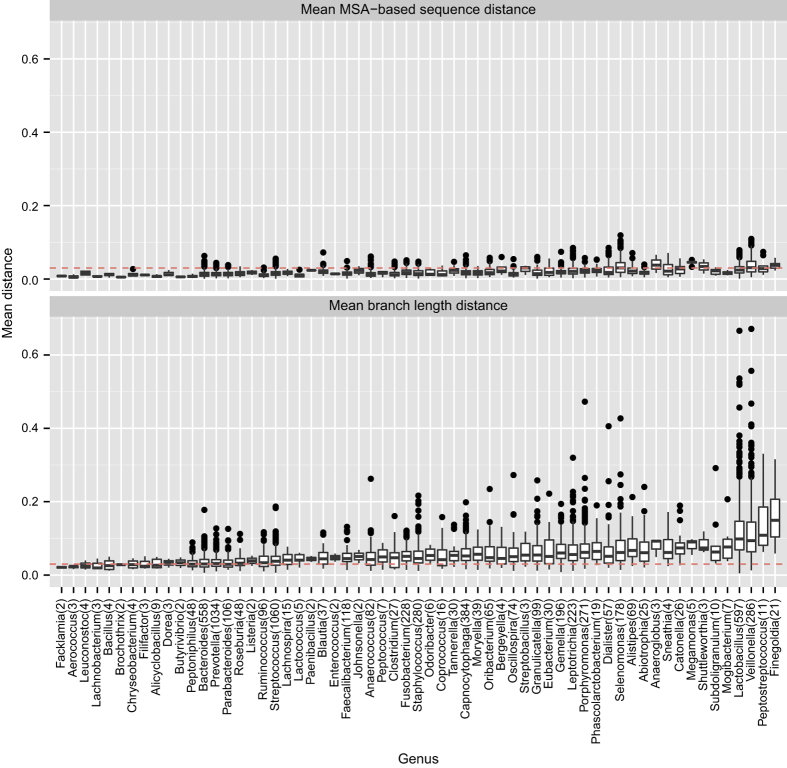
Box plots of the mean MSA and BLD across the different genera within the Firmicutes, Bacteroidete, and Fusobacteria phyla for the V1V3 OTU. We report the distribution of the mean MSA-based sequence dissimilarity and mean phylogenetic branch length distance between the representative sequence and all other sequences within an OTU for the V1V3 OTUs. We group the distributions according to the OTUs’ genus-level annotations. The red line delineates a distance of 0.03. We report the total number of OTUs that belong to that genus in parenthesis.

Even within well-known and well-studied phyla such as Bacteroidetes, Firmicutes, and Fusobacteria, we still see large variations across OTUs (Figure 3). For example, both *Staphylococcus* and *Lactobacillus* are two genera with many OTUs from Firmicutes. The 280 V1V3 OTUs that belonged to *Staphylococcus* were very tightly clustered: the mean MSD and BLD were 0.017 and all the OTUs within *Staphylococcus* carried the same annotation as the representative sequence. The 597 V1V3 OTUs that belonged to *Lactobacillus*, on the other hand, had nearly double the average mean distances (MSD and BLD of 0.026 and 0.118), with OTU 7767 having a mean MSD of 0.050 and mean BLD of 0.519, greatly eclipsing the average MSD and BLD distances for the V1V3 OTUs. The 2.0% of the V1V3 OTUs from *Lactobacillus* contained sequences with annotations that differed from the representative sequence, including OTU 7767.

**Figure 4:**
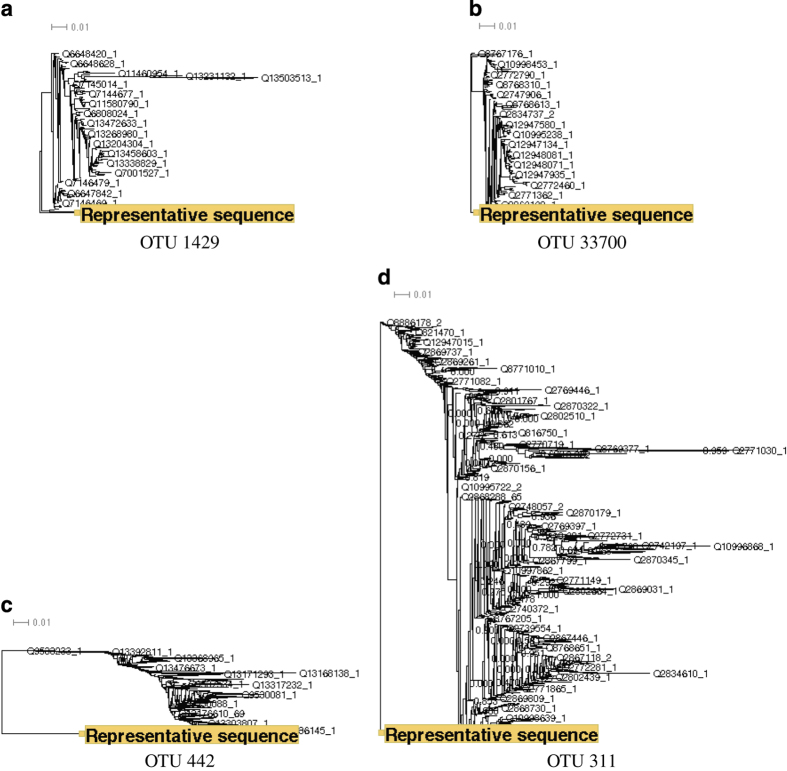
ML trees for the V1V3 OTUs classified as the genus *Sneathia*. We show the ML trees estimated on the V1V3 OTU alignments that belong to the genus *Sneathia*. The OTUs are (**a**) OTU 1429, (**b**) OTU 33700, (**c**) OTU 442, and (**d**) OTU 311. The representative sequence for each OTU is highlighted in yellow. All trees are drawn on the same scale.

Other examples of poorly clustered OTUs from a well-studied phylum are the V1V3 OTUs from the genus *Sneathia* (belonging to the phylum Fusobacteria). There are a total of four V1V3 OTUs from *Sneathia*, however, these OTUs had on average the highest mean BLD compared with all other genera. Half of the *Sneathia* OTUs could have different annotations, depending on which sequence was selected as the representative. The ML trees estimated on the four OTU alignments from *Sneathia* varied greatly in shape (Figure 4). For example, the representative sequence from OTU 1429 (the yellow sequence in Figure 4a) was on a very long branch and seemed to cluster separately from the majority of the remaining sequences. OTU 33700 (Figure 4b), on the other hand, was very compact, and contained very short branches. OTU 311 (Figure 4d) showed the most variation, with several distinct clusters of sequences. These results are indicative that using PSD may be missing the true diversity of the microbiome as the OTUs are being over clustered.

**Figure 5:**
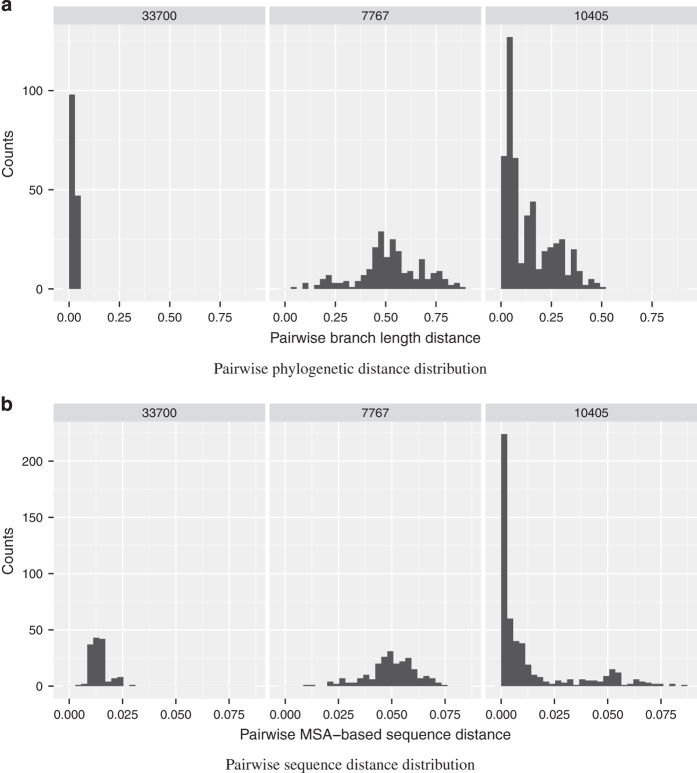
Distribution of the MSA and BLD for the V1V3 OTUs 33700, 10405, 7767. We show the (**a**) pairwise phylogenetic branch length distributions and (**b**) MSA-based sequence dissimilarity distributions for three V1V3 OTUs. OTU 33700 belongs to the genus *Sneathia*, OTU 7767 belongs to the genus *Lactobacillus*, and OTU 10405 belongs to the phylum TM7.

To highlight the variation we saw across the taxonomic tree, we showed the distribution of the MSD and BLD for three V1V3 OTUs: OTU 33700 from the genus *Sneathia*, OTU 7767 from the genus *Lactobacillus*, and OTU 10405 from the phylum TM7 (Figure 5). These OTUs displayed very different characteristics. As previous mentioned, OTU 33700 had low-mean pairwise distances; the distributions of both the MSD and BLD for the OTU were very compact and each had a single peak. OTU 7767, on the other hand, had much broader distributions. Interestingly, while OTU 7767’s MSD distribution seemed to follow a normal distribution, the BLD seemed more like a mixture of normal distributions. Finally, OTU 10405’s distributions showed even more divergent behaviors, with each distribution containing potentially three peaks.

Although we only showed three distributions from our analyses, similar trends can be seen across the different taxonomic groups. We hypothesize that these large variations in distributions are caused by the use of PSD for computing OTUs. We see that the OTUs defined by PSD are consistent when the evolutionary distance between sequences is low, such as for *Staphylococcus*. However, when the true evolutionary distance is much larger, such as for V1V3 OTUs from *Sneathia*, PSD is overestimating the similarity between sequences. This can have profound impact for downstream analyses. For example, *Sneathia* can be part of the regular microbiota in the vaginal microbiome in human females; however, *Sneathia* is also correlated with various medical conditions including preterm labor and bacteria vaginosis.^19^ Grouping *Sneathia* sequences with 97% similarity based upon PSD may result in inaccurate clinical diagnosis as pathogenic strains may be missed due to over clustering.

One interesting observation is that the candidate phylum TM7 showed the largest diversity for the V1V3 OTUs. This suggests that perhaps we need to take into account the properties of the sequences, such as where the sequences belong in the taxonomic tree, in selecting a threshold. Indeed, many new methods have been developed to cluster sequences using variable thresholds.^20–23^ Other techniques like oligotyping and minimum entropy decomposition might aid in OTU analyses by examining the variation within an OTU.^12,24^ More comprehensive studies comparing these different approaches are needed, however, in order to move the community toward using more sophisticated methods to obtain improved OTU clustering.

These results highlight the importance of using accurate metrics for estimating the similarity between sequences. The original QIIME study used optimal pairwise sequence alignments to compute the percent similarity (i.e., the PSD) that was used to generate the OTUs. However, we saw that using PSD resulted in poorly clustered OTUs, even though all the PSD within an OTU were above the 97% similarity threshold. When we use sequence dissimilarity distances based upon a MSA (i.e., the MSD), we find that >80% of the OTUs actually contained sequences that were below the sequence similarity threshold. However, the MSD still underestimates the total amount of evolution as the phylogenetic branch length distance was on average more than three times that of the MSA-based sequence dissimilarity distance. This suggests that we need to shift toward using evolutionary-based distance metrics in order to build OTU bins that contain sequences that are evolutionarily similar to the representative sequences.

Another alternative to sequence similarity-based clustering is phylogeny-based OTU clustering.^25^ Rather than grouping sequences based upon sequence similarity, we can use placement algorithms^26–28^ to insert the reads into a reference tree and then cluster sequences using phylogenetic distances between the reads. Phylogeny-based OTU binning would take into account a model of sequence evolution and might lead to more accurate clusters.

More concerning are the results from annotating every read in the data set. One-tenth to one-fifth of the OTUs (depending on the region used) could have different annotations depending on which sequence was selected as the representative sequence. As OTUs are commonly used to estimate the diversity of a microbial sample, two analyses of the same data set can lead to different conclusions. For example, the abundance estimations for a particular community could be biased depending on which sequences were selected as the representative sequences, as well as which region was amplified.

Perhaps these results suggest that we should move beyond using OTUs. OTUs, as currently used in most analyses, are an artificial construct that makes our analyses computationally easier. However, being easy to run does not equate to being accurate. If we are only interested in comparing clusters of sequences, then perhaps current OTU clustering methods are sufficient. If we are interested in taxonomic identification, then we should strive toward using methods that classify every read rather than only looking at representative sequences.

This perspective paper presents a cautionary tale for OTU clustering analyses. We saw that PSD, the commonly used metric for computing sequence dissimilarity distances, resulted in poorly clustered data, and that using a more accurate sequence-based metric like MSD could detect cases in which the similarity between sequences was being overestimated. However, we also saw that the MSD was still underestimating the evolutionary distances between sequences. We saw that the poorly clustered OTUs grouped divergent sequences, and that the classification of an OTU could vary depending on which sequence was selected as the representative member. We as a community need to begin discussing the problems with using OTUs. Only then can we shift the paradigm from using the traditional, but potentially misleading methods, to more accurate and phylogeny-based methods.

## Materials and methods

### 16S data sets

The sequence data sets used in this study were obtained from the Human Microbiome Project QIIME Community Profiling website (http://hmpdacc.org/HMQCP/). This webpage includes the QIIME analyses on all the 16S V1V3 and V3V5 samples from the NCBI SRA projects SRP002395: Human Microbiome Project 16S rRNA Clinical Production Phase I and SRP002012: Human Microbiome Project 454 Clinical Production Pilot. From the webpage, we downloaded the QIIME-binned OTUs (binned at 97% sequence similarity using USEARCH,^29^ the final OTU annotation table, and the representative sequences for each OTU. The details for generating the OTUs are provided on the HMP QIIME Community Profiling webpage.^30^

### Phylogenetic analysis

We downloaded all high-quality full-length 16S sequences taken from type isolates from the RDP database.^31^ A reference alignment was estimated on the RDP sequences using PASTA version 1.6.3.^32^ We produced a reference tree by using the NCBI taxonomy on the reference sequences as a constraint tree and refined the NCBI taxonomy into a binary tree using RAxML version 8.1.3 (ref. 33) on the PASTA alignment.

We selected all OTUs with at least 100 sequences (29,020 OTUs in total; 12,326 V1V3 OTUs and 16,694 V3V5 OTUs) and aligned each OTU to the reference alignment using UPP 2.0,^34^ a fast and accurate method for aligning fragmentary sequences to an existing reference alignment. For each OTU alignment, we computed the MSD of the representative sequence to all other sequences within the OTU. Note that the MSD was computed on the UPP alignment generated by aligning all the OTU sequences to the 16S reference alignment rather than estimating a *de novo* MSA for each OTU. This technique of reference-based MSA allows us to align millions of reads independently of each other and makes it trivial to parallelize the alignment step.

We estimated an ML tree on the OTU alignment using FastTree-2 version 2.1.7,^35^ a fast and accurate method for estimating ML trees on very large MSAs. We computed the BLD between the representative sequence and all other sequences within the OTU using the ML tree.

The perl script used to compute the MSD for a given alignment and the R script used to compute the BLD for a given tree are available at https://github.com/namphuon/phylo.

### 16S annotation

We followed the protocol used in the HMP QIIME pipeline to annotate the 16S sequences. We ran RDP classifier version 2.2^31^ using the greengenes^36^ training input files provided by QIIME (ftp://greengenes.microbio.me/greengenes_release/gg_13_5/gg_13_5_otus.tar.gz). We report the percentage of OTUs that had a sequence that is classified differently from the representative sequence. A query sequence has a differing classification if it is under classified (i.e., representative sequence classified at genus level and the query sequence is classified at the family level), over classified (i.e., representative sequence classified at family level and the query sequence is classified at the genus level), or conflicting (i.e., the representative sequence is classified as genus A and the query sequence is classified as genus B).

### Commands

- PASTA—run pasta.py -i *<*input file*>*
- RAxML—raxmlHPC-PTHREADS -m GTRGAMMA -T 8 -p 1111 -g *<*unrefined taxonomic tree*>* -s *<*pasta alignment*>* -n refine
- UPP—python run upp.py -f *<*input file*>* -o *<*output prefix*>* -m dna -t *<*pasta tree*>* -a *<*pasta alignment*>* -A 100
- FastTree—fasttree -gtr -gamma -nt *<*input alignment*> > <*output tree*>*
- RDP classifier—assign taxonomy.py -i *<*input otu*>* -r gg 97 otus 4feb2011.fasta -t green- genes tax rdp train.txt -o *<*output file*>* -m rdp
